# Parental Early Life Maltreatment and Related Experiences in Treatment of Youth Anxiety Disorder

**DOI:** 10.1007/s10578-023-01520-1

**Published:** 2023-03-20

**Authors:** Thomas B. Bertelsen, Bente Storm Mowatt Haugland, Gro Janne Wergeland, Åshild Tellefsen Håland

**Affiliations:** 1 Department of Child and Adolescence Mental Health, Sørlandet Sykehus, Egsveien 100, 4630 Kristiansand, Norway; 2 Department of Clinical Child and Adolescent Psychology, Faculty of Psychology, University of Bergen, Bergen, Norway; 3 Department of Clinical Psychology, University of Bergen, Bergen, Norway; 4 Division of Psychiatry, Department of Child and Adolescent Psychiatry, Haukeland University Hospital, Bergen, Norway; 5 Department of Clinical Medicine, Faculty of Medicine, University of Bergen, Bergen, Norway

**Keywords:** Parental early life maltreatment, Parental depressive symptoms, Anxiety, Youth anxiety, Cognitive behavioral therapy

## Abstract

The role of parents’ early life maltreatment (ELM) (e.g. physical, sexual abuse) and related experiences, in relation to offspring anxiety is not well understood. The current study investigated the association between self-reported depression and ELM and related experiences in mothers (*n* = 79) and fathers (*n* = 50), and mother-, father-, and youth-reported symptoms of youth anxiety (*n* = 90). Outcomes were assessed at pre,- and posttreatment and 3-, 6-, and 12-months follow-up. Parental ELM were not associated with pre-treatment differences or differences in outcome of treatment. However ELM related experiences were associated with increased mother-, father-, and youth-rated youth anxiety at pretreatment. Fathers depressive symptoms were found to mediate the relationship between father ELM related experiences and father-rated youth anxiety symptoms. Future research is warranted on parental ELM and depression as factors affecting outcomes of treatment of youth anxiety. Trial registered at: helseforskning.etikkom.no (reg. nr. 2017/1367).

## Introduction

An estimated thirty-one percent of adults have experienced early life maltreatment (ELM), which includes sexual, physical, or emotional abuse in childhood and adolescent years (≤ 18 years) [1]. Such experiences have long-lasting consequences and are associated with an increased risk of later physical and psychiatric symptoms and disorders [2–4]. Beyond the effects such abuse has on the victim, ELM has been hypothesized to negatively impact the mental health of descendants as well [5]. It has been reported that ELM increases the risk of internalizing disorders among offspring of victims [6–8]. However, there is limited knowledge on how parental ELM affects offspring anxiety disorders. Such information may have important implications for the understanding and treatment of anxiety disorders in youth.

The role of parental ELM in offspring’s mental health is considered both environmental and (epi-)genetic. The psychological impacts of ELM could influence parenting practices [9]. Specifically, ELM has been found to be associated with more authoritarian and permissive parenting, as well as aggressive parenting behavior, which may account for important environmental effects of parental ELM on youths’ mental health [10, 11]. Alternatively, the negative effects of ELM on the mental health of offspring may be explained by epigenetic factors, such as altered glucocorticoid genes in parents that are passed on to their offspring [12]. Among several plausible explanations for the link between parental ELM and offspring psychopathology, the best evidenced is the role of mother psychological distress, which may negatively influence offspring development through epigenetic changes, difficulties with attachment, and poor parenting practices [13]. The importance of mother psychological distress does not imply that fathers are unimportant, but rather reflects that there is a paucity of research on the link between father ELM and offspring psychopathology.

Several studies have found ELM to be a risk factor for depression in adults [14–16]. Depression in adults is considered to negatively impact youth offspring anxiety [17, 18]. The connection between parental depression and youth anxiety has been related to withdrawn, intrusive, or inconsistent parenting styles, which may potentially create an insecure environment for youth [19]. In line with this, several studies indicate an association between parental ELM and offspring anxiety symptoms, which is mediated by parental depressive symptoms [6, 14].

Because parental ELM may influence parents' mental health and parenting practices, it is plausible that ELM also negatively affects treatment outcomes for child anxiety. Parental psychopathology has been found to negatively affect outcomes of CBT for child and adolescent anxiety [20]. Specifically, parental depression has been found to affect outcomes of CBT for child anxiety [21]. However, this association has not been found consistently and is affected by factors such as child age [20]. In particular it has been found that adolescents have poorer parent-reported rate of change, when their parent is depressed [22].

Despite evidence to support a connection between parent ELM and offspring anxiety, there is a paucity of studies that have assessed parental ELM in clinical youth and on outcomes of treatment. Only three studies investigate parental ELM in youth referred for clinical treatment [15, 23, 24]. Two studies by Miranda et al. [15, 23] investigated the association between mother’s childhood abuse (psychological, physical, and/or sexual abuse) and youth diagnostic status, clinician-rated youth functioning, and mother-rated youth behavioral and emotional problems. Their sample included 547 youth who had not yet begun treatment (age range: 8–17, *M*_*age*_ = 13.3, 46% female) recruited from psychiatric outpatient settings. Their findings suggest that mother ELM was associated with maternal psychopathology and child externalizing difficulties. Another study investigated the association between maternal childhood trauma and parenting style in mothers of youth receiving mental health services [24]. Their sample consisted of 95 youths who had not yet begun treatment (*M*_*age*_ = 15.1, 46% female) recruited from a range of public youth mental health services. Their findings suggested that mothers ELM was associated with less child reported maternal acceptance and higher degrees of controlling behaviour.

Although laudable for addressing a topic of clinical significance, these studies did not investigate whether parental ELM affected outcomes of youth mental health treatment. Such knowledge is important since the presence of parental ELM may have important implications for understanding and treating youth mental in general, and youth anxiety in particular. Also, there is a paucity of studies assessing the effect of fathers ELM related to offspring mental health [13]. Those studies that have examined fathers ELM in relation to offspring mental health have either used a highly specific definition of traumatic events (i.e., World Trade Center and the holocaust [7, 8]) or have limited definitions of youth mental health (i.e., using a single question asked to parents about whether their child has a certain disorder [5]). Furthermore, exploring differences between mother and father ELM may be of particular importance in youth anxiety, because youth anxiety is believed to be differentially affected by mothers and fathers [25–27].

In the current study, we assess associations between mother and father ELM and youth anxiety symptoms, clinical impairment, and treatment outcomes in a sample of 90 families referred to treatment for youth anxiety. Both mothers and fathers were assessed for the presence of ELM. Multiple informants were used to assess youth anxiety symptoms and clinical impairment (mother, father, youth, clinician). Parental ELM was assessed as a predictor of treatment outcomes over a 12-month study period. Our first research aim was to investigate the associations between paternal or maternal ELM and youth anxiety symptoms and clinical impairment from anxiety across informants at pretreatment. Our second aim was to investigate the effect of fathers or mothers ELM on their youth’s treatment outcomes. For treatment outcomes predicted by parental ELM, we aimed to investigate the mediating role of parental depression between parental ELM and anxiety outcomes.

## Methods

### Participants and Procedure

The study sample was based on a clinical trial evaluating the outcomes of a CBT program named RISK [28]. The treatment was delivered in a multi-family group format, with five to eight families per group. The treatment consisted of 12 sessions, lasting 38 h administered over 10 weeks. In two of these sessions school personnel (i.e., teachers, school nurses) attended. A main component of the treatment was to facilitate in-session and out-of-session exposure practice. The sample comprised 90 youths, 79 mothers, and 50 fathers. The youths were between 12 and 18 years old (*M*_age_ = 15.3 years, SD = 1.3; 76.5% female). The treatment was designed to include all types of anxiety disorders, including obsessive–compulsive disorder (OCD). Inclusion criteria were meeting the diagnostic criteria for any anxiety disorder or OCD as determined by the ADIS-IV [29]. Participants were excluded if intellectual disability, autism spectrum disorder, ongoing self-harm, suicidal ideation, or psychotic disorder were present. Additionally, participants could not receive concurrent psychological treatment and had to attend school for more than 50% of the time over the previous month. Youth receiving psychopharmacological treatment were included if the medication had been stable for at least 6 months. Exclusion based on school attendance was due to practical concerns about the treatment which involved school personnel. Written informed consent was obtained from both youth and parents, and the Regional Ethics Committee approved the study (reg. nr. 2017/1367).

Participants were recruited from ordinary referrals to two community-based clinics for child and adolescent mental health. Both are part of the general national health service in Norway. Trained clinicians conducted diagnostic interviews to determine the youths’ diagnostic status (see Sect. "Dependent Measures").

Youth, mothers, and fathers reported on anxiety symptoms at pretreatment, posttreatment, and at 3-, 6-, and 12-months follow-up. Clinicians reported on clinical global impression at pretreatment, posttreatment, and at 3-, 6-, and 12-months follow-up. Clinical severity rating based on diagnostic interview was assessed at pretreatment and posttreatment and 12-month follow-up. Since the diagnostic interview was not conducted at 3-, and 6-month follow-up, a last-observation carried forward procedure was used as a conservative estimate of youth diagnostic status and clinical severity rating at these timepoints [30].

### Measures

#### Dependent Measures

The ADIS-IV-C/P [29] was used to determine the youths’ diagnostic status and clinical severity ratings (CSR). This is a semi-structured diagnostic interview administered separately to youth and parents. Diagnoses and CSR were assigned according to the ADIS-IV-C/P manual. A CSR of four or higher (0–8 scale) indicates the presence of a diagnosis. In this study, the CSR of youth’s primary diagnosis was applied as a measure of overall diagnostic impairment. The ADIS-IV-C/P has excellent reliability [31]. Diagnostic interviews were videotaped, and independent evaluators reassessed 20%, randomly selected interviews. Inter-rater reliability on diagnostic severity was excellent, with Cronbach’s α ranging from 0.91 to 0.97.

The Spence Children’s Anxiety Scale (SCAS; [32], child and parent versions, were used to assess youth anxiety symptoms. The SCAS was completed by youth (SCAS-C, *n* = 90), mothers (SCAS-M; *n* = 79), and fathers (SCAS-F; *n* = 50). The SCAS includes 38 items rated on a 4-point, yielding a maximum total score of 114. The SCAS has shown good psychometric properties [33]. Internal consistency (Cronbach’s *α*) in the current sample was good to excellent (Child *α* = 0.90; Mother *α* = 0.85, Father *α* = 0.71).

The severity measure of the Clinical Global Impression—Severity (CGI-S) [34] scale was used to assess clinician-rated global impairment and functioning. The CGI-S evaluates the severity of the patient’s illness and comprises seven items ranging from 1 (*normal*) to 7 (*extremely ill*). The CGI-S is significantly correlated with self-reported measures of anxiety, depression, everyday functioning, and quality of life [35]. In this study, the CGI-S showed excellent reliability (split-half coefficient = 0.92).

#### Predictors and Mediating Variables

Parental ELM was assessed at pretreatment using two questions derived from the family of origin subscale of the Systemic Therapy Inventory of Change (STIC) [36]. These questions asked about the occurrence of the following: a. having experienced sexual abuse within the family, b. having experienced physical abuse within the family. All questions were answered on a 5-point scale ranging from 0 (“Never”) to 4 (“All the time”). Before answering questions respondents were given the following prompt: “Please select the alternative that best describes the family you grew up with as a child or youth. Choose the period of your childhood that best describes your experiences with your family”. In this study, we assessed the presence of any ELM based on whether parents had scores higher than 0 on any question. Parents experiencing any ELM was 15% and 5% of mothers and fathers, respectively. Questions and descriptive statistics can be found in Table 1.

**Table 1:** Descriptive statistics for parental early life maltreatment and related experiences

Question	Mother				Father			
	*M*	SD	*n*	Present	*M*	SD	*n*	Present
Someone in my family abused medication or illegal drugs	0.38	0.44	76	13%	0.25	0.86	49	11%
Someone in my family abused alcohol	0.92	1.29	78	39%	0.78	1.16	49	37%
Someone in my family exhibited inappropriate sexual behavior or made sexual advances towards other family members	0.15	0.58	78	8%	0.06	0.32	49	4%
I felt threatened by/was afraid of someone in my family	0.53	1.05	78	24%	0.18	0.49	49	14%
Someone in my family used physical force to get what they wanted	0.36	0.84	78	18%	0.10	0.37	49	8%
Someone in my family used inappropriate physical force or hit me	0.26	0.75	68	13%	0.10	0.45	39	5%
Someone in my family sexually abused me	0.13	0.52	68	7%	0	0	39	0%
Total	2.29	3.65	79	50%	0.78	1.73	50	25%

The Patient Health Questionnaire depression scale (PHQ-9; [37] was used to assess parental depression symptoms. The PHQ-9 assess depressive symptoms during the last 2 weeks. The PHQ-9 was completed separately by mothers (*n* = 79) and fathers (*n* = 50). The PHQ-9 comprises 9 items rated on a 4-point scale, with a maximum total score of 27. The PHQ-9 has demonstrated good psychometric properties [37]. Internal consistency in the current sample was good (Mother *α* = 0.84; Father *α* = 0.86).

Early sampling experiences indicated that people who anecdotally had experienced ELM did not wish to respond to the ELM questions, but reported other negative experiences in their childhood. To include these participants secondary analyses were performed on a more inclusive and less restrictive operationalization of ELM, which we termed ELM and Related behaviour (ELM-R). ELM-R was assessed with the STIC [36] using the two questions from ELM as well as five questions asking for the occurrence of the following: c. substance abuse in the close family, d. alcohol abuse in the close family, e. fearing one’s own family, f. having seen sexual abuse within the family, and g. having seen physical abuse within the family. It is important to note that ELM-R does not imply presence of ELM, but constitute risks for the presence of ELM. Thus, although abuse of alcohol or substances does not imply child maltreatment, they may still be viewed as important risk factors for child maltreatment [38]. Parents experiencing ELM-R was 50% and 25% for mothers and fathers.

### Data Analytic Strategy

Data was gathered as part of a study assessing the effect of family involvement in CBT for youth anxiety [28], and sample size was based on power calculations for that outcome study. Prior to analysing the data in the current study, sensitivity power analyses were performed using G*power version 3.1 [39]. As recommended by Faul et al. (2007) sensitivity power analyses were conducted to assess what effect sizes could be detected in the current study. On tests of pretreatment differences between youth of parents with or without ELM/ELM-R, the power to detect an effect of Cohens *d* = 0.6 was 0.80 and 0.50 for mothers and fathers, respectively. In regressions to assess the influence of parental ELM/ELM-R on child treatment outcomes, the power to detect an effect of Cohens *d* = 0.3 was 0.80 and 0.50 for mothers and fathers, respectively. Based on recommendations from [40] we expected to have 0.80 power to detect mediation pathways larger than Cohen’s *d* = 0.4. Given the relatively small sample of father’s assessments and the risk of committing type II errors, we included Bayesian analyses for all tests involving the effects of father ELM/ELM-R. These analyses indicate which hypothesis is most reasonable when findings are nonsignificant and help clarify nonsignificant results in low-powered studies [41].

Pretreatment differences were assessed using independent samples t-tests. The effect of the presence of parental ELM on treatment outcomes was assessed using a multi-level model in the R package lme4 [42], R Core Team [43]. Multilevel models were used because they allow the specification of nested structures (repeated time for the same individual within a family) and flexibly handle missing information [44]. Mediation analyses were performed in JASP using the mediation module [45]. For all analyses, parental ELM was dichotomized to indicate the presence or absence of any type of ELM. Multilevel and mediation models were controlled for the effect of baseline clinical severity.

Given that multiple comparisons were planned we adjusted the analyses using Benjamini–Hochberg procedures, which ensure that the family-wise error risk remains at the specified level (i.e., 0.05) despite multiple comparisons [46]. The Benjamini–Hochberg procedure was preferred over alternative correction methods, such as the Bonferroni-correction, to reduce the risk of type II errors.

To aid the interpretation of potential differences in outcomes between informants, the intra-class correlation (ICC) was calculated as a measure of inter-rater agreement [47]. Inter-rater agreement was good between CSR1 and CGI-S (κ = 0.72) and fair between SCAS-C and SCAS-M (κ = 0.53). On all other outcomes inter-rater agreement was poor (κ < 0.40).

## Results

Correlations for all analyzed variables can be found in Table 2. Littles MCAR test indicated that the data were different from missing completely at random (*p* < 0.05). Further analysis suggested the data to be missing at random with high levels of ELM-R predicting missing on ELM. Therefore missing data was handled using full-information maximum likelihood were applicable. The five questions specific to ELM-R were significant predictors of presence of ELM and could correctly classify 98% of mothers and fathers as having or not having experienced ELM. Results suggested significant correlations between SCAS-C, SCAS-M, and mother ELM-R. PHQ-M was significantly correlated with SCAS-M and mother ELM-R, whereas PHQ-F was significantly correlated with SCAS-F and father ELM-R.

**Table 2:** Descriptive statistics study measures at pretreatment

Variable	*n*	*M*	SD	1	2	3	4	5	6	7	8	9	10	11
1. SCAS-C	90	42.4	15.3	–										
2. SCAS-M	79	29.3	17.3	0.50***	–									
3. SCAS-F	50	15.9	16.6	− 0.02	0.20	–								
4. CGI-S	90	6.6	1.1	0.01	0.05	0.13	–							
5. CSR1	90	5.3	0.8	0.12	0.02	0.12	0.35***	–						
6. PHQ-M	79	4.8	4.3	0.13	0.60***	− 0.04	0.05	0.06	–					
7. PHQ-F	50	2	3.6	− 0.06	0.07	0.53***	0.01	0.01	0.12	–				
8. ELM-RM	79	0.5	–	0.35***	0.36***	0.01	0.01	0.00	0.23*	0.09	–			
9. ELM-RF	50	0.25	–	− 0.14	0.03	0.51***	0.06	0.00	− 0.14	0.33***	− 0.02	–		
10. ELM-M	79	0.15	–	0.24*	0.06	− 0.21	0.02	− 0.05	− 0.03	− 0.24*	0.38***	− 0.25*	–	
11. ELM-F	50	0.05		− 0.19	0.11	0.05	0.02	− 0.04	0.12	− 0.13	− 0.20	0.28	− 0.08	–

*SCAS* Spence Children’s Anxiety Scale, *C* child, *M* mother, *F* father, *PHQ* Patient Health Questionnaire, *ELM-R* Early Life Maltreatment Related experiences, *ELM* Early Life Maltreatment, *CGI-S* Clinical Global Impression—Severity, *CSR1*, Clinical Severity Rating of primary diagnosis as assessed by ADIS-IV-C/P

*p < 0.05. **p < 0.01 ***p < 0.001

Our first research question was whether mother and father ELM would result in higher ratings of child anxiety symptoms and functional impairment at pretreatment. Children whose mothers had experienced ELM rated significantly higher on SCAS-C [*t*(66) = 1.96, *p* = 0.049, *d* = 0.65], but significantly lower on the CGI [*t*(68) = 3.12, *p* < 0.001, *d* = 0.99]. On the measures SCAS-F, SCAS-M and CSR there were no difference between mothers with or without ELM (*p* > 0.05). Children whose fathers had experienced ELM were not different at pre-treatment on any outcome measures (*p* > 0.05). Overall, the results indicated that children of mothers with ELM regarded themselves as having more anxiety symptoms than those of mothers without ELM. Surprisingly, clinicians rated children of mothers with ELM as less severe.

Our second research question was whether mother and father ELM would reduce the effectiveness of treatment. As shown in Table 3, there was no effect of either mother or father ELM on changes during treatment in any outcome variables.

**Table 3:** Parental Early Life Maltreatment as predictor of treatment outcome for youth anxiety (*N* = 90)

Dependent	Predictor	Beta	SE	95% CI		*p*	Bayes Factor
				LL	UL		
CSR							
	Time	− 0.91	0.09	− 1.09	− 0.73	< 0.0001	
	ELM-F	− 0.37	1.41	− 3.13	2.39	0.79	< 0.0001
	ELM-M	0.12	1.23	− 2.29	2.53	0.92	
	Time*ELM-F	0.21	0.32	− 0.42	0.84	0.51	2.74
	Time*ELM-M	0.12	0.31	− 0.49	0.73	0.29	
CGI-S							
	Time	− 0.68	0.07	− 0.82	− 0.54	< 0.0001	
	ELM-F	− 0.76	1.03	− 2.78	1.26	0.46	0.02
	ELM-M	− 1.42	0.87	− 3.13	0.29	0.11	
	Time*ELM-F	0.15	0.27	− 0.38	0.68	0.58	0.03
	Time*ELM-M	0.39	0.25	− 0.10	0.88	0.12	
SCAS-C							
	Time	− 3.69	0.56	− 4.79	− 2.59	< 0.0001	
	ELM-F	− 0.40	8.77	− 17.59	16.79	0.96	< 0.0001
	ELM-M	0.06	7.28	− 14.21	14.33	0.99	
	Time*ELM-F	− 1.27	2.38	− 5.93	3.39	0.59	0.11
	Time*ELM-M	0.13	1.91	− 3.61	3.87	0.95	
SCAS-F							
	Time	− 5.27	0.75	− 6.74	− 3.80	< 0.0001	
	ELM-F	− 3.52	10.46	− 24.02	16.98	0.74	< 0.0001
	ELM-M	− 5.41	8.63	− 22.32	11.50	0.53	
	Time*ELM-F	1.37	2.87	− 4.26	7.00	0.63	< 0.0001
	Time*ELM-M	0.87	2.38	− 3.79	5.53	0.72	
SCAS-M							
	Time	− 3.48	0.73	− 4.91	− 2.05	< 0.0001	
	ELM-F	− 0.48	10.53	− 21.12	20.16	0.96	< 0.0001
	ELM-M	− 18.64	8.68	− 35.65	− 1.63	0.03	
	Time*ELM-F	− 0.52	2.76	− 5.93	4.89	0.85	< 0.0001
	Time*ELM-M	2.31	2.29	− 2.18	6.80	0.31	

The Bayes Factor represents the weight of evidence in favor of *H*_*1*_ divided by the weight of evidence in favor of *H*_*0*_*.* The Bayes Factor was used for outcomes on father reports given relatively low statistical power

*SCAS* Spence Children’s Anxiety Scale, *C* child, *M* mother, *F* father, *PHQ* Patient Health Questionnaire, *ELM* Early Life Maltreatment, *CGI-S* Clinical Global Impression—Severity, *CSR1* Clinical Severity Rating of primary diagnosis as assessed by ADIS-IV-C/P, *LL* lower limit, *UL* upper limit

### Secondary Analyses

Children whose mothers had Early Life Maltreatment Related experiences (ELM-R) rated significantly higher at pre-treatment on SCAS-C [*t*(77) = 3.27, *p* = 0.002, *d* = 0.75] and SCAS-M [*t*(77) = 3.63, *p* < 0.001, *d* = 0.76], but there were no differences regarding SCAS-F, CGI, and CSR (*p* > 0.05). However, children whose fathers had experienced ELM-R had significantly higher scores at pre-treatment on the SCAS-F [*t*(48) = 5.62, *p* < 0.001, *d* = 1.35] but not on the other outcome measures (i.e., SCAS-C, SCAS-M, CGI, CSR). As shown in Table 4, there was no effect of either mother or father ELM-R on changes during treatment in CSR or CGI-S. In line with the findings on pretreatment scores, mother ELM-R showed significant interactions with changes in SCAS-C and SCAS-M. This indicates that mother ELM-R reduced the effectiveness of treatment. In addition, father ELM-R showed significant interactions with time with regard to SCAS-F.

**Table 4:** Parental Early Life Maltreatment Related experiences as predictor of treatment outcome for youth anxiety (*N* = 90)

Dependent	Predictor	Beta	SE	95% CI		*p*	Bayes Factor
				LL	UL		
CSR							
	Time	− 0.89	0.05	− 0.99	− 0.79	< 0.0001	
	ELMR-F	0.22	0.24	− 0.25	0.69	0.36	0.001
	ELMR-M	− 0.06	0.22	− 0.49	0.37	0.77	
	Time*ELM-RF	0.04	0.05	− 0.06	0.14	0.49	2.41
	Time*ELM-RM	− 0.01	0.05	− 0.11	0.09	0.81	
CGI-S							
	Time	− 0.64	0.05	− 0.74	− 0.54	< 0.0001	
	ELMR-F	− 0.01	0.18	− 0.36	0.34	0.99	< 0.0001
	ELM-MR	0.04	0.16	− 0.27	0.35	0.80	
	Time*ELM-RF	0.03	0.05	− 0.07	0.13	0.60	< 0.0001
	Time*ELM-RM	0.01	0.05	− 0.09	0.11	0.76	
SCAS-C							
	Time	− 4.38	0.42	− 5.20	− 3.56	< 0.0001	
	ELMR-F	− 0.30	1.47	− 3.18	2.58	0.84	0.35
	ELMR-M	− 2.18	1.37	− 4.87	0.51	0.11	
	Time*ELM-RF	0.32	0.42	− 0.50	1.14	0.45	0.54
	Time*ELM-RM	1.54	0.38	0.80	2.28	< 0.0001	
SCAS-F							
	Time	− 3.93	0.42	− 4.75	− 3.11	< 0.0001	
	ELMR-F	− 8.79	1.61	− 11.95	− 5.63	< 0.0001	> 1000
	ELMR-M	1.19	1.49	− 1.73	4.11	0.98	
	Time*ELM-RF	1.60	0.42	0.78	2.42	< 0.0001	292
	Time*ELM-RM	− 0.24	0.38	− 0.98	0.50	0.53	
SCAS-M							
	Time	− 4.19	0.49	− 5.15	− 3.23	< 0.0001	
	ELMR-F	− 0.13	1.89	− 3.83	3.57	0.94	0.02
	ELMR-M	3.75	1.75	0.32	7.18	0.03	
	Time*ELM-RF	0.01	0.49	− 0.95	0.97	0.98	< 0.001
	Time*ELM-RM	1.32	0.44	0.46	2.18	< 0.0001	

The Bayes Factor represents the weight of evidence in favor of *H*_*1*_ divided by the weight of evidence in favor of *H*_*0*_*.* The Bayes Factor was used for outcomes on father reports given relatively low statistical power

*SCAS* Spence Children’s Anxiety Scale, *C* child, *M* mother, *F* father, *PHQ* Patient Health Questionnaire, *ELM* Early Life Maltreatment, *ELM-R* Early Life Maltreatment Related experiences, *CGI-S* Clinical Global Impression—Severity, *CSR1* Clinical Severity Rating of primary diagnosis as assessed by ADIS-IV-C/P, *LL* lower limit, *UL* upper limit

### Exploratory Analyses

To understand a potential mechanism of how parental ELM affected treatment outcomes, we investigated the mediating role of mother and father depression. These analyses were only performed on significant interactions, which meant only ELM-R variables were included. Thus these analyses should be considered exploratory. As seen in Table 5, maternal depression did not significantly mediate the relationship between mother ELM-R and SCAS-C or SCAS-M. However, father depression did significantly mediate the relationship between father ELM-R and SCAS-F.

**Table 5:** Mediation analyses

Mediation pathway	Unstandardized estimate	SE	95% CI		Standardized estimate	SE	*p*
ELM-RM −> PHQ-M −> SCAS-C	0.35	0.35	− 0.33	1.03	0.02	0.02	0.32
ELM-RF −> PHQ-F −> SCAS-F	1.96	0.82	0.37	3.56	0.14	0.06	0.02
ELM-RM −> PHQ-M −> SCAS-M	1.18	1.15	− 1.06	3.43	0.07	0.07	0.30

*SCAS* Spence Children’s Anxiety Scale, *C* child, *M* mother, *F* father, *PHQ* Patient Health Questionnaire, *ELM-R* Early Life Maltreatment Related experiences, *CGI* Clinical Global Impression, *CSR1* Clinical Severity Rating of primary diagnosis as assessed by ADIS-IV-C/P, *LL* Lower limit, *UL* upper limit

## Discussion

The current study did not find parental Early Life Maltreatment (ELM) to affect outcomes of treatment on clinical ratings (CGI) or diagnostic outcomes (CSR). Surprisingly, clinicians rated youth with mothers who experienced ELM less severe at pre-treatment. The current study found youth-reported anxiety symptoms at pre-treatment to be higher in youth whose mothers had experienced early life maltreatment and related experiences (ELM-R). Mothers ELM-R was found to reduce the effect of treatment on youth anxiety symptoms rated by mothers and youth themselves. Fathers’ ELM-R was found to reduce the effect of treatments on father-rated youth anxiety symptoms. Exploratory analyses suggests that fathers depression mediates the relationship between fathers ELM-R and fathers rating of youth anxiety symptoms during treatment.

The finding that third-party raters (here clinicians) do not observe negative differences between children of parents with or without ELM agrees with previous studies [6, 14]. This may be interpreted as parents with ELM are more tuned-in to symptoms of anxiety in their children and thus are better able to assess the presence of these. Alternatively, parents that have experienced ELM may be overly sensitive to their child’s symptom presentation and thus perceiving symptoms exaggerated [6]. A supporting finding of the interpretation that mothers with ELM are better able to assess youth anxiety is the finding that these mothers had the highest inter-rater agreement with youth self-reported anxiety, whereas other ratings of youth anxiety had poor levels of agreement.

The presence of mother ELM-R predicted poorer treatment outcomes regarding both mother and youth-rated anxiety symptoms, whereas father ELM-R only predicted poorer outcomes on father-rated youth anxiety symptoms. The importance of mother ELM-R and its effect on youth anxiety may reflect associations between ELM-R and authoritarian, permissive, and aggressive parenting behaviors, potentially creating an insecure upbringing environment for youths [10, 11]. However, such associations could equally predict father ELM-R to affect youth anxiety. The discrepancy between the effect of mother and father ELM-R may be due to cultural practices where mothers spend more time with children and thus exert greater environmental influence over their development. Alternatively, the unique impact of mother ELM-R may be due to epigenetic mechanisms transferred in utero [48]. Such a mechanism is in line with previous research indicating altered stress reactions in the offspring of mothers with ELM-R [49, 50]. According to this line of research, mother ELM-R plays a unique role due to epigenetic mechanisms transferred in utero [48].

Although findings suggest father ELM and ELM-R to be less influential than mother ELM-R, the importance of fathers should not be discounted based on current research. Previous research has shown father ELM to be uniquely associated with offspring anxiety [7], and in relation to CBT the fathers' role has been highlighted, particularly for older children [25, 51]. The special role of fathers may be due to evolutionary adaptions causing instinctual reliance on fathers for feedback on the external environment [51]. Thus, youth anxiety may be particularly affected by father signals of danger, which may also be affected by fathers’ ELM. Given the theoretical importance of fathers in relation to youth anxiety it is important to note that the current study had low power to detect the effects of fathers. Despite the low informativeness of the results, we believe it is important to further investigate the role of fathers’ ELM in youth anxiety due to the paucity of pre-existing research.

Father depression was found to mediate the effect of father ELM-R on the fathers rating of their child's anxiety symptoms. This could be seen as a further argument for the idea that parents’ ratings of child anxiety reflect the ratings of their own mental health symptoms. This could be understood as typical cognitive biases found in individuals with depression, who tend to view themselves and others from a pessimistic perspective. However, this finding could be due to environmental effects of the parents depression that may affect internalizing disorders of youth, such as guilt induction or interparental conflict [52, 53].

The strengths of the current study are the assessment of both mother and father ELM, the multi-informant strategy, and the context of a treatment study with documented effectiveness. Despite these advantages, the current study contains certain limitations. The first limitation is that ELM was assessed by self-report, using specific questions from STIC [36]. Although the STIC is psychometrically well-established [36], there is no previous research on the validity of the questions selected in this study to assess parental ELM. Related to this limitation, the questions defined as ELM-R have not previously been established as predictive of ELM and should only be interpreted as *risk* factors (i.e., substance and alcohol abuse) for ELM and not ELM per se. However, the validity of the ELM-R construct is in part confirmed by the finding that all parents with ELM reported ELM-R, and ELM-R predicted ELM with 98% accuracy. An additional limitation of the reporting of ELM is that self-report may have biased results due to recall bias or potential underreporting of ELM. This limitation may also have affected mediation models because of recall bias such that depressed fathers having a negative perception of their child and their own childhood. A second limitation is the small sample used to analyse ELM and related lower power to detect effects. This limitation is in part addressed by the secondary and exploratory analyses, which include a larger sample using the ELM-R construct. Despite the small sample size, we believe that the results are important, given the paucity of research on the relationship between parent ELM and youth anxiety. Finally, the current sample was selected based on youth referred to treatment for their anxiety disorder, and thus findings may not generalize to other settings.

In conclusion, parental ELM and ELM-R was not associated with diagnostic or functional improvement. Father ELM-R was associated with higher father-reported youth anxiety symptoms at pre-treatment. Mother ELM was associated with higher youth-reported anxiety symptoms at pre-treatment. Mother ELM-R was associated with higher mother-, and youth-reported youth anxiety symptoms. Furthermore, mother and father ELM-R predicted lesser treatment gains on measures of anxiety symptoms. The relationship between father ELM-R and father ratings of anxiety symptoms during treatment, was mediated by fathers depression. The clinical implications of these findings are that it may be beneficial to assess and address parents’, early life experiences and current depressive symptoms as these may affect the treatment outcomes of youth anxiety negatively. Further research is needed to understand the mechanisms of the relationship identified in the present study.

## Summary

The role of parents’ early life maltreatment (e.g. physical, sexual abuse) and related experiences, in relation to offspring anxiety is not well understood. The current study investigated the association between self-reported depression and ELM and related experiences (e.g., fear, substance abuse) in mothers (*n* = 79) and fathers (*n* = 50), and mother-, father-, and youth-reported symptoms of youth anxiety (*n* = 90). The study was carried out in a clinical setting, which treated youth with an anxiety disorder. The treatment was a multifamily group exposure therapy. Outcomes were assessed at pre,—and posttreatment and 3-, 6-, and 12-months follow-up. Parental ELM were not associated with pre-treatment differences or differences in outcome of treatment. However, ELM related experiences were associated with increased mother-, father-, and youth-rated youth anxiety at pretreatment. Additionally, mother ELM related experiences affected outcomes of treatment on youth-, and mother-rated youth anxiety. Likewise father ELM related experiences affected outcomes of treatment on father-rated youth anxiety. Furthermore, fathers’ depressive symptoms were found to mediate the relationship between father ELM related experiences and father-rated youth anxiety symptoms. Future research is warranted on parental ELM and depression as factors affecting outcomes of treatment of youth anxiety.

## References

[1] Witt A, Brown RC, Plener PL, Brähler E, Fegert JM (2017) Child maltreatment in Germany: prevalence rates in the general population. Child Adolesc Psychiatry Ment Health 11:47. 10.1186/s13034-017-0185-0 28974983 10.1186/s13034-017-0185-0 PMC5621113

[2] Afifi TO, Mather A, Boman J, Fleisher W, Enns MW, Macmillan H, Sareen J (2011) Childhood adversity and personality disorders: results from a nationally representative population-based study. J Psychiatr Res 45(6):814–822. 10.1016/j.jpsychires.2010.11.008 21146190 10.1016/j.jpsychires.2010.11.008

[3] Goodwin RD, Stein MB (2004) Association between childhood trauma and physical disorders among adults in the United States. Psychol Med 34(3):509–520. 10.1017/s003329170300134x 15259836 10.1017/s003329170300134x

[4] Hovens JGFM, Wiersma JE, Giltay EJ, van Oppen P, Spinhoven P, Penninx BWJH, Zitman FG (2010) Childhood life events and childhood trauma in adult patients with depressive, anxiety and comorbid disorders vs. controls. Acta Psychiatr Scand 122(1):66–74. 10.1111/j.1600-0447.2009.01491.x 19878136 10.1111/j.1600-0447.2009.01491.x

[5] Arditti R, Strat YL (2020) A traumatic life experience in childhood increases the risk of a psychiatric disorder in the offspring. Psychiatry Res 290:113101. 10.1016/j.psychres.2020.113101 32474066 10.1016/j.psychres.2020.113101

[6] Morrel TM, Dubowitz H, Kerr MA, Black MM (2003) The effect of maternal victimization on children: a cross-informant study. J Fam Violence. 10.1023/A:1021401414414

[7] Yehuda R, Bell A, Bierer LM, Schmeidler J (2008) Maternal, not paternal, PTSD is related to increased risk for PTSD in offspring of Holocaust survivors. J Psychiatr Res 42(13):1104–1111. 10.1016/j.jpsychires.2008.01.002 18281061 10.1016/j.jpsychires.2008.01.002 PMC2612639

[8] Yehuda R, Halligan SL, Bierer LM (2001) Relationship of parental trauma exposure and PTSD to PTSD, depressive and anxiety disorders in offspring. J Psychiatr Res 35(5):261–270. 10.1016/s0022-3956(01)00032-2 11591428 10.1016/s0022-3956(01)00032-2

[9] Lange BCL, Callinan LS, Smith MV (2019) Adverse childhood experiences and their relation to parenting stress and parenting practices. Community Ment Health J 55(4):651–662. 10.1007/s10597-018-0331-z 30194589 10.1007/s10597-018-0331-z PMC6447511

[10] Leslie LA, Cook ET (2015) Maternal trauma and adolescent depression: is parenting style a moderator? Psychology 06(06):681–688. 10.4236/psych.2015.66066

[11] Newcomb MD, Locke TF (2001) Intergenerational cycle of maltreatment: a popular concept obscured by methodological limitations. Child Abuse Negl 25(9):1219–1240. 10.1016/s0145-2134(01)00267-8 11700694 10.1016/s0145-2134(01)00267-8

[12] Youssef NA, Lockwood L, Su S, Hao G, Rutten BPF (2018) The effects of trauma, with or without PTSD, on the transgenerational DNA methylation alterations in human offsprings. Brain Sci. 10.3390/brainsci8050083 29738444 10.3390/brainsci8050083 PMC5977074

[13] Plant DT, Pawlby S, Pariante CM, Jones FW (2018) When one childhood meets another—maternal childhood trauma and offspring child psychopathology: a systematic review. Clin Child Psychol Psychiatry 23(3):483–500. 10.1177/1359104517742186 29171287 10.1177/1359104517742186

[14] Koverola C, Papas MA, Pitts S, Murtaugh C, Black MM, Dubowitz H (2005) Longitudinal investigation of the relationship among maternal victimization, depressive symptoms, social support, and children’s behavior and development. J Interpers Violence 20(12):1523–1546. 10.1177/0886260505280339 16246915 10.1177/0886260505280339

[15] Miranda JK, de la Osa N, Granero R, Ezpeleta L (2013) Maternal childhood abuse, intimate partner violence, and child psychopathology: the mediator role of mothers’ mental health. Violence Against Women 19(1):50–68. 10.1177/1077801212475337 23386668 10.1177/1077801212475337

[16] Springer KW, Sheridan J, Kuo D, Carnes M (2007) Long-term physical and mental health consequences of childhood physical abuse: results from a large population-based sample of men and women. Child Abuse Negl 31(5):517–530. 10.1016/j.chiabu.2007.01.003 17532465 10.1016/j.chiabu.2007.01.003 PMC3031095

[17] Hirshfeld-Becker DR, Micco JA, Henin A, Petty C, Faraone SV, Mazursky H, Bruett L, Rosenbaum JF, Biederman J (2012) Psychopathology in adolescent offspring of parents with panic disorder, major depression, or both: a 10-year follow-up. Am J Psychiatry 169(11):1175–1184. 10.1176/appi.ajp.2012.11101514 23534056 10.1176/appi.ajp.2012.11101514

[18] Reeb BT, Wu EY, Martin MJ, Gelardi KL, Shirley Chan SY, Conger KJ (2015) Long-term effects of fathers’ depressed mood on youth internalizing symptoms in early adulthood. J Res Adolesc 25(1):151–162. 10.1111/jora.12112 25750495 10.1111/jora.12112 PMC4350018

[19] Langrock AM, Compas BE, Keller G, Merchant MJ, Copeland ME (2002) Coping with the stress of parental depression: parents’ reports of children’s coping, emotional, and behavioral problems. J Clin Child Adolesc Psychol 31(3):312–324. 10.1207/S15374424JCCP3103_03 12149969 10.1207/S15374424JCCP3103_03

[20] Knight A, McLellan L, Jones M, Hudson J (2014) Pre-treatment predictors of outcome in childhood anxiety disorders: a systematic review. Psychopathol Rev a1(1):77–129. 10.5127/pr.034613

[21] Southam-Gerow MA, Kendall PC, Weersing VR (2001) Examining outcome variability: correlates of treatment response in a child and adolescent anxiety clinic. J Clin Child Adolesc Psychol 30(3):422–436. 10.1207/s15374424jccp3003_13 10.1207/S15374424JCCP3003_13 11501258

[22] Legerstee JS, Huizink AC, van Gastel W, Liber JM, Treffers PD, Verhulst FC, Utens EM (2008) Maternal anxiety predicts favourable treatment outcomes in anxiety-disordered adolescents. Acta Psychiatr Scand 117(4):289–298. 10.1111/j.1600-0447.2008.01161.x 18321354 10.1111/j.1600-0447.2008.01161.x

[23] Miranda JK, de la Osa N, Granero R, Ezpeleta L (2011) Maternal experiences of childhood abuse and intimate partner violence: psychopathology and functional impairment in clinical children and adolescents. Child Abuse Negl 35(9):700–711. 10.1016/j.chiabu.2011.05.008 21903266 10.1016/j.chiabu.2011.05.008

[24] Zalewski M, Cyranowski JM, Cheng Y, Swartz HA (2013) Role of maternal childhood trauma on parenting among depressed mothers of psychiatrically ill children. Depress Anxiety 30(9):792–799. 10.1002/da.22116 23649503 10.1002/da.22116 PMC4536924

[25] Bögels S, Phares V (2008) Fathers’ role in the etiology, prevention and treatment of child anxiety: a review and new model. Clin Psychol Rev 28(4):539–558. 10.1016/j.cpr.2007.07.011 17854963 10.1016/j.cpr.2007.07.011

[26] Connell AM, Goodman SH (2002) The association between psychopathology in fathers versus mothers and children’s internalizing and externalizing behavior problems: a meta-analysis. Psychol Bull 128(5):746–773. 10.1037/0033-2909.128.5.746 12206193 10.1037/0033-2909.128.5.746

[27] Liber JM, van Widenfelt BM, Goedhart AW, Utens EMWJ, van der Leeden AJM, Markus MT, Treffers PDA (2008) Parenting and parental anxiety and depression as predictors of treatment outcome for childhood anxiety disorders: has the role of fathers been underestimated? J Clin Child Adolesc Psychol 37(4):747–758. 10.1080/15374410802359692 18991126 10.1080/15374410802359692

[28] Bertelsen TB, Wergeland GJ, Nordgreen T, Himle JA, Håland ÅT (2022) Benchmarked effectiveness of family and school involvement in group exposure therapy for adolescent anxiety disorder. Psychiatry Res 313:114632. 10.1016/j.psychres.2022.114632 35597139 10.1016/j.psychres.2022.114632

[29] Silverman WK, Albano AM (1996) Anxiety disorder interview schedule for DSM-IV. Child version. Oxford University Press Inc., Oxford

[30] Siddiqui O, Hung HMJ, O’Neill R (2009) MMRM vs. LOCF: a comprehensive comparison based on simulation study and 25 NDA datasets. J Biopharm Stat 19(2):227–246. 10.1080/10543400802609797 19212876 10.1080/10543400802609797

[31] Silverman WK, Saavedra LM, Pina AA (2001) Test-retest reliability of anxiety symptoms and diagnoses with the anxiety disorders interview schedule for DSM-IV: child and parent versions. J Am Acad Child Adolesc Psychiatry 40(8):937–944. 10.1097/00004583-200108000-00016 11501694 10.1097/00004583-200108000-00016

[32] Spence SH (1998) A measure of anxiety symptoms among children. Behav Res Ther 36(5):545–566. 10.1016/s0005-7967(98)00034-5 9648330 10.1016/s0005-7967(98)00034-5

[33] Arendt K, Hougaard E, Thastum M (2014) Psychometric properties of the child and parent versions of Spence children’s anxiety scale in a Danish community and clinical sample. J Anxiety Disord 28(8):947–956. 10.1016/j.janxdis.2014.09.021 25445085 10.1016/j.janxdis.2014.09.021

[34] Guy W (1976) ECDEU assesment manual for psychopharmacology. Department of Health, Education, and Welfare Public Health Service Alcohol, Drug Abuse, and Mental Health Administration

[35] Zaider TI, Heimberg RG, Fresco DM, Schneier FR, Liebowitz MR (2003) Evaluation of the clinical global impression scale among individuals with social anxiety disorder. Psychol Med 33(4):611–622. 10.1017/s0033291703007414 12785463 10.1017/s0033291703007414

[36] Pinsof WM (2017) The systemic therapy inventory of change—STIC: a multi-systemic and multi-dimensional system to integrate science into psychotherapeutic practice. In: Tilden T, Wampold BE (eds) Routine outcome monitoring in couple and family therapy: the empirically informed therapist. Springer, Cham, pp 85–101. 10.1007/978-3-319-50675-3_5

[37] Kroenke K, Spitzer RL, Williams JB (2001) The PHQ-9: validity of a brief depression severity measure. J Gen Intern Med 16(9):606–613. 10.1046/j.1525-1497.2001.016009606.x 11556941 10.1046/j.1525-1497.2001.016009606.x PMC1495268

[38] Stith SM, Liu T, Davies LC, Boykin EL, Alder MC, Harris JM, Som A, McPherson M, Dees JEMEG (2009) Risk factors in child maltreatment: a meta-analytic review of the literature. Aggress Violent Behav 14(1):13–29. 10.1016/j.avb.2006.03.006

[39] Faul F, Erdfelder E, Lang A-G, Buchner A (2007) G*Power 3: a flexible statistical power analysis program for the social, behavioral, and biomedical sciences. Behav Res Methods 39(2):175–191. 10.3758/BF03193146 17695343 10.3758/bf03193146

[40] Fritz MS, Mackinnon DP (2007) Required sample size to detect the mediated effect. Psychol Sci 18(3):233–239. 10.1111/j.1467-9280.2007.01882.x 17444920 10.1111/j.1467-9280.2007.01882.x PMC2843527

[41] Dienes Z (2014) Using Bayes to get the most out of non-significant results. Front Psychol 5:781. 10.3389/fpsyg.2014.00781 25120503 10.3389/fpsyg.2014.00781 PMC4114196

[42] Bates D, Mächler M, Bolker B, Walker S (2014) Fitting linear mixed-effects models using lme4. arXiv preprint arXiv:1406.5823

[43] R Core Team R (2020) R: a language and environment for statistical computing. [Computer software]. R Foundation for Statistical Computing

[44] Hayes AF (2006) A primer on multilevel modeling. Hum Commun Res 32(4):385–410. 10.1111/j.1468-2958.2006.00281.x

[45] JASP JT (2020) JASP (0.14) [computer software]

[46] Benjamini Y, Hochberg Y (1995) Controlling the false discovery rate: a practical and powerful approach to multiple testing. J R Stat Soc: B 57(1):289–300. 10.1111/j.2517-6161.1995.tb02031.x

[47] Cicchetti DV (1994) Guidelines, criteria, and rules of thumb for evaluating normed and standardized assessment instruments in psychology. Psychol Assess 6(4):284–290. 10.1037/1040-3590.6.4.284

[48] Yahyavi ST, Zarghami M, Marwah U (2014) A review on the evidence of transgenerational’ ’ transmission of posttraumatic stress disorder vulnerability. Revista brasileira de psiquiatria (Sao Paulo, Brazil: 1999) 36(1):89–94. 10.1590/1516-4446-2012-0995 24402183 10.1590/1516-4446-2012-0995

[49] Danielson CK, Hankin BL, Badanes LS (2015) Youth offspring of mothers with posttraumatic stress disorder have altered stress reactivity in response to a laboratory stressor. Psychoneuroendocrinology 53:170–178. 10.1016/j.psyneuen.2015.01.001 25622009 10.1016/j.psyneuen.2015.01.001 PMC4333024

[50] Liu K, Ruggero CJ, Goldstein B, Klein DN, Perlman G, Broderick J, Kotov R (2016) Elevated cortisol in healthy female adolescent offspring of mothers with posttraumatic stress disorder. J Anxiety Disord 40:37–43. 10.1016/j.janxdis.2016.04.003 27088877 10.1016/j.janxdis.2016.04.003 PMC4964788

[51] Bögels S, Perotti EC (2011) Does father know best? A formal model of the paternal influence on childhood social anxiety. J Child Fam Stud 20(2):171–181. 10.1007/s10826-010-9441-0 21475711 10.1007/s10826-010-9441-0 PMC3048306

[52] Fear JM, Champion JE, Reeslund KL, Forehand R, Colletti C, Roberts L, Compas BE (2009) Parental depression and interparental conflict: children and adolescents’ self-blame and coping responses. J Fam Psychol 23(5):762–766. 10.1037/a0016381 19803612 10.1037/a0016381 PMC4244839

[53] Rakow A, Forehand R, McKee L, Coffelt N, Champion J, Fear J, Compas B (2009) The relation of parental guilt induction to child internalizing problems when a caregiver has a history of depression. J Child Fam Stud 18(4):367–377. 10.1007/s10826-008-9239-5 20090863 10.1007/s10826-008-9239-5 PMC2808109

